# Reducing Viral Load Measurements to Once a Year in Patients on Stable, Virologically Suppressive Cart Regimen: Findings from the Australian HIV Observational Database

**DOI:** 10.4172/2155-6113.1000383

**Published:** 2014-12

**Authors:** Mahshid Rafiee, Azar Kariminia, Stephen Wright, Graham Mills, Ian Woolley, Don Smith, David J. Templeton, Matthew G. Law, Kathy Petoumenos

**Affiliations:** 1 The Kirby Institute, UNSW Australia, Sydney, NSW, Australia; 2 Department of Medicine, Waikato Hospital, Hamilton, New Zealand; 3 Infectious Diseases Department, Monash Medical Centre, Vic., Australia; Department of Medicine, Monash University, Vic., Australia; 4 The Albion Street Centre, Sydney, NSW, Australia; School of Public Health and Community Medicine, UNSW Australia, Sydney, NSW, Australia; 5 The Kirby Institute, UNSW Australia, Sydney, NSW, Australia; RPA Sexual Health, Sydney Local Health District, Sydney, NSW, Australia; Central Clinical School, University of Sydney, Sydney, NSW, Australia

**Keywords:** HIV, Antiretroviral therapy, HIV viral load, HIV viral load monitoring, HIV resistance

## Abstract

Reducing viral-load measurements to annual testing in virologically suppressed patients increases the estimated mean time those patients remain on a failing regimen by 6 months. This translates to an increase in the proportion of patients with at least one Thymidine Analogue Mutation from 10% to 32% over one year.

## Introduction

In the current era of effective treatment, reducing the frequency of virological monitoring may become more common. Current treatment guidelines recommend viral load monitoring every 3 to 4 months in clinically stable patients with suppressed viral load [1]. However, studies have previously indicated that viral load monitoring may be safely reduced to 6-monthly in stable patients [2]. There is little data on the impact of reducing viral load monitoring to annually, yet anecdotal evidence from Australia suggests that some clinicians are extending the interval between viral load measures for up to one year in clinically stable and virologically suppressed HIV positive (HIV+) patients.

We aimed to investigate the effects of reducing the frequency of viral load tests to annually among HIV^+ve^ patients with long-term virological control.

## Methods

We used data from the Australian HIV Observational Database (AHOD). Patients were required to fulfil the following inclusion criteria: commenced combination Antiretroviral Therapy (cART) on or after 1 January 1997; remained virologically suppressed (<400 copies/mL) while on a stable cART regimen for at least one year; and had two or more viral load measurements per year.

Person-year methods were used to calculate the rate of virological failure (defined as two consecutive detectable viral loads (≥400 copies/mL) within one year or one measure of virological failure followed by a change of treatment within one year). Baseline date was the end of the first year of experiencing suppressed viral load while on a stable regimen. Follow-up was calculated from baseline to the time of virological failure; or (a) the date treatment was stopped/interrupted for more than 14 days or (b) the last visit date for patients who did not fail (censored).

To estimate the additional time a patient remained on a failing regimen if HIV viral load testing occurred annually we created a paired dataset by duplicating patient data and allowing each patient to act as his/her own control. The first line of data in each pair included all the viral load measures and the true stop or failure date from the observed data. The second line included a theoretical annual HIV test date calculated as the anniversary date of the baseline date. The patients’ censor or failure date was therefore the last anniversary date from baseline that was greater or equal to the observed true stop or failure date. We calculated the additional time on a failing regimen as the time to failure using the observed data subtracted from the theoretical data.

We estimated the rate of accumulation of Nucleoside Reverse Transcriptase Inhibitor (NRTI), non- Nucleoside Reverse Transcriptase Inhibitor (NRTI) and Thymidine Analogue Mutation (TAM) resistance mutations if the rate of viral load testing was reduced to annual testing. Estimates were based on the rates of resistance accumulated in patients remaining on failing regimens as reported by Sigaloff et al. [3] and Cozzi-Lepri et al. [4]. We assumed the rate of resistance mutations accumulated exponentially and that virological failures occur uniformly in relation to viral load testing. Hence, if viral load testing was carried out annually, 25% of failures fail in the period 0–3 months after the previous viral load test, a further 25% in the period 3–6 months, 25% during 6–9 months and the final 25% during the period 9–12 months since the last viral load test.

To illustrate the absolute impact of reduced viral load testing we applied the failure rate reported in AHOD to a hypothetical population of 1000 HIV patients who had been virologically suppressed on cART for one year, and subsequently followed for two years. We estimated the number of patients who would be expected to fail virologically based on AHOD data, the reduced number of viral load tests over the two years if only annual virological monitoring, and contrast that with the increase in proportion of failing patients who develop resistance during the two-year period.

## Results

By March 2013, 3551 patients were recruited to AHOD, of whom 2651 started cART on or after 1 January 1997; 584 (16%) patients fulfilled our study inclusion criteria. Most were male (92%) with overall mean age of 50 years (SD: 11.2). The median viral load at cART initiation was 68 200 copies/ml (IQR, 17 347–149 000).

Overall 76 patients (13%) experienced virological failure over 1946 person-years (py) of follow-up with a failure rate of 3.9 per 100 py. Of the failures 64 (84%) switched treatment following two consecutive failures while 12 patients (16%) switched treatment after a single measurement of >400 copies/ml. The mean extra time patients remained on a failing regimen if viral load testing is reduced to annual was estimated at 6 months (SD: 3.1).

Table 1 shows the rate of accumulated resistance based on the rates reported by Sigaloff et al. [3] and Cozzi Lepri et al. [4]. If the frequency of viral load monitoring remained at every 3 months, then based on the Sigaloff data, we estimate that 10% would have TAM related resistance, while 4.4% would be NRTI-related and 7% NNRTI-related. If viral load monitoring was performed annually and we assumed that of patients who failed 25% did so in each of the windows 0–3, 3–6, 6–9 and 9–12 months following failure, then the estimated rate of resistance accumulated over one year would be 32% with at least one TAM, and 16% and 35% with any NRTI and NNRTI mutation respectively.

Based on the data from Cozzi-Lepri et al. [4] we estimated that with 3-monthly viral load monitoring 3% of failing patients would have accumulated one or more TAMs, compared with an estimated 11% of failing patients with annual viral load monitoring.

In our hypothetical population of 1000 patients, if we applied the failure rate observed in AHOD (3.9/100 py) we estimate that 80 patients would have failed within two years. Regular three monthly viral load testing would yield approximately 9000 viral load tests over a complete 2-year period (including tests at baseline and at year 2). Reducing viral load testing to annually would reduce the viral load tests to 3000, a saving of around 6000 viral load tests over two years. However, based on the data from Sigaloff et al. [3] the reduction in viral load testing to annually would increase the rate of accumulated TAMs in the 80 patients who failed from 10% to 32%.

## Discussion

We reported a relatively high rate of virological failure of almost 4 per 100 py among virologically suppressed patients in AHOD. Reducing viral load testing to annually increased the time patients remained on a failing regimen for an additional 6 months. While this translates to 6000 less viral load tests over two years, the proportion of the 80 failures with at least one TAM could increase from 10% to 32% (applying the rates reported by Sigaloff et al. [3]); or from 3% to 11% according to the Cozzi-Lepri estimates.

There is considerable variation in rates of developing antiretroviral resistance mutations in the published literature [5,6]. The lower estimate produced from the Cozzi-Lepri et al. [4] is based on patients from the EuroSIDA cohort and may more likely reflect the rates in other developed countries, including Australia. Although reducing viral load testing frequency may be cost-effective, as demonstrated in the case of reducing the frequency of CD4 monitoring [7], we have shown a high failure rate even among patients on apparently stable suppressive therapy. If viral load testing was reduced then around one-third of these patients who fail could potentially develop further resistance mutations, thereby reducing potential future treatment choices. Our results contrast with the findings by Reekie et al. who found that it is reasonable to extend visit intervals to 6 months for stable and fully suppressive patients on cART with minimal impact on treatment failure 3 to 6 months later [2].

There are some limitations to our analysis. First, we have used >400 copies for the virological failure cutoff as the data spanned over 10 years of data where many of the AHOD sites will still using assays at this level. However, using a higher viral load level in some ways may indicate true virological failures rather than virological blips that might range between 50 and 400 copies/mL. Second, we censored patients with treatment interruptions of greater than 14 days. We therefore would have excluded some patients who had one measure of virological failure and who stopped treatment, therefore still failing off treatment and subsequently accumulating resistance. Thirdly, we assumed that patients with virological failure were still taking some of their cART, thereby maintaining selection pressure for the evolution of resistance mutants. If these patients had discontinued all cART, ongoing viral evolution may not be occurring. Finally, we are unable to determine whether reducing the frequency of viral load testing would decrease the frequency of attendance or have an effect on adherence in this model.

Despite effective and well-tolerated regimens, stable and suppressed patients continue to fail therapy. While annual viral monitoring could reduce virological testing costs, our study suggests there could be a substantial increase in the numbers of failing patients who develop resistance which has its own costs due to more expensive resistance testing, treatment regimens and potential community costs of increased transmission of drug resistance virus. The pros and cons of reduced frequency of viral load monitoring in stable patients warrants more research, including well-conducted randomised trials.

## Figures and Tables

**Table 1:** Probabilities of accumulated mutation for virological failure.

Studies	Mutation	Rate of accumulation (per month) (95% CI)	1 Probabilities of accumulated mutations				
			0–3 month window	3–6 month window	6–9 month window	9–12 window month	At one year
**Sigaloff et al.** [3]	TAM^2^	0.07 (0.04–0.11)	0.1	0.27	0.4	0.52	0.32
	NRTI-associated	0.03 (0.01–0.06)	0.04	0.13	0.2	0.27	0.16
	Any NNRTI-associated	0.05 (0.03–0.09)	0.07	0.2	0.31	0.41	0.25
**Cozzi-Lepri et al.** [4]	Any TAM mutation	0.02^3^	0.03	0.09	0.14	0.19	0.11

1 We assumed virological failure distributed uniformly during one year, 25% of failures fail in the period 0–3 months after the previous viral load test, a further 25% in the period 3–6 months, 25% during 6–9 months and the final 25% during the period 9–12 months since the last viral load test.

2 Thymidine Analogue Mutation (TAM).

3 Yearly rate was given in the Cozzi_Lepri, et al. study and therefore confidence interval for monthly rate cannot be calculated.
